# Supplementary figures and images for: JIP4 and RILPL1 utilize opposing motor force to dynamically regulate lysosomal tubulation
Source: J Cell Biol. 2025 Sep 24;224(11):e202404018. doi: 10.1083/jcb.202404018 (PMC12459091; doi:10.1083/jcb.202404018)

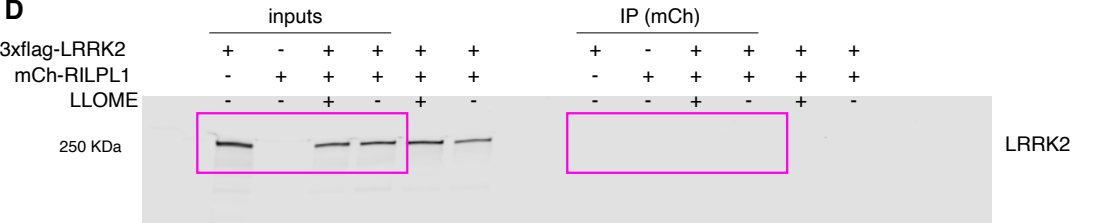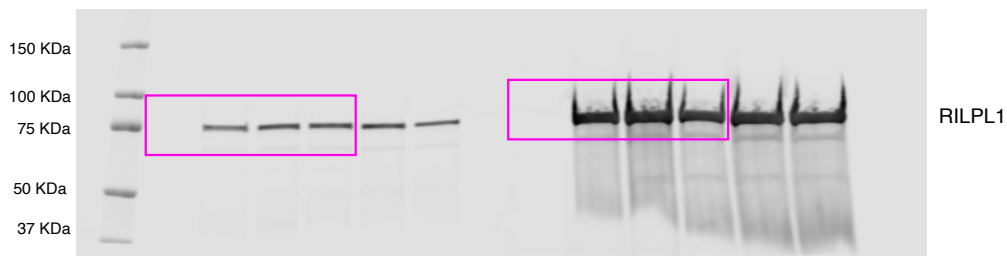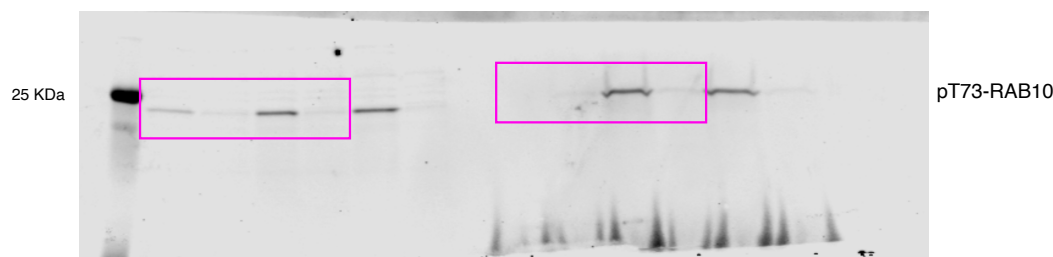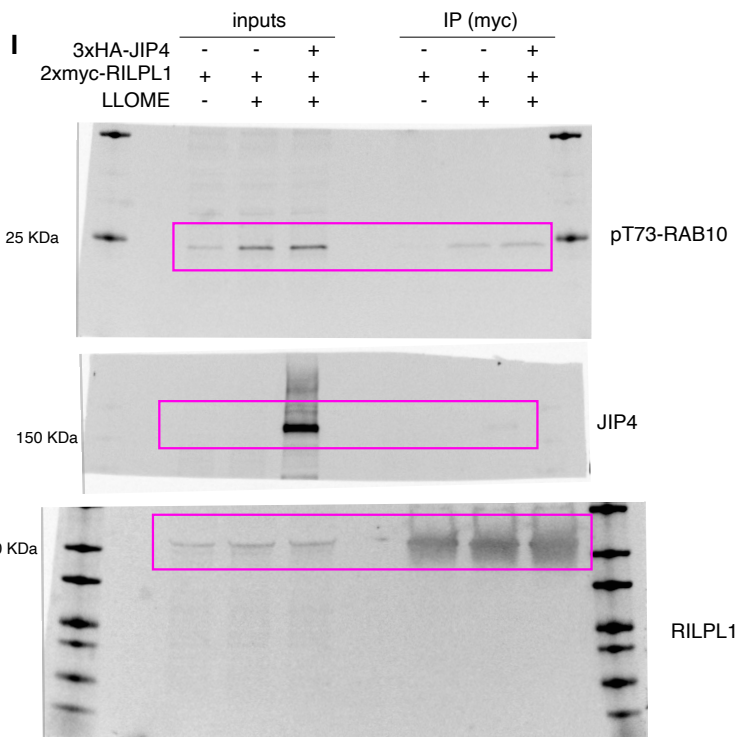

Supplement: SourceData F3 — is the source file for Fig. 3. [file jcb_202404018_sourcedataf3.pdf]

**F**

NTC      siDYNC1H1

250 KDa

DYNC1H1

50 KDa

 $\alpha$ -tubulin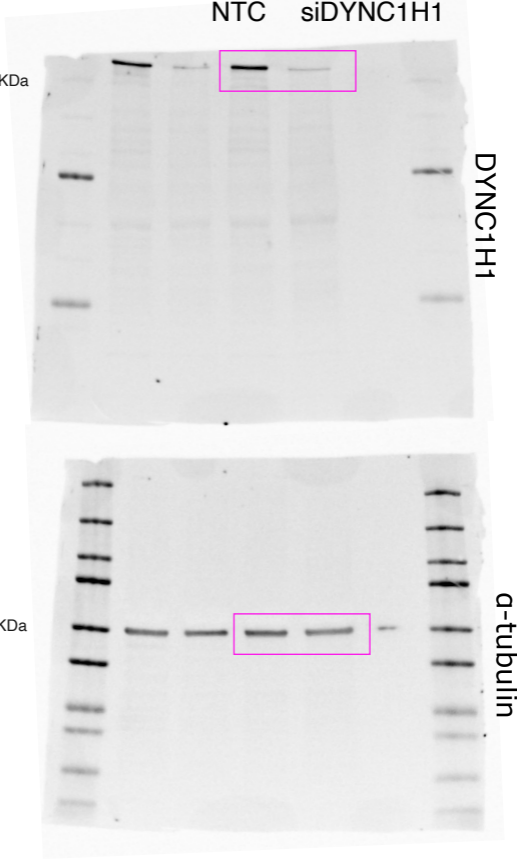

Supplement: SourceData F4 — is the source file for Fig. 4. [file jcb_202404018_sourcedataf4.pdf]

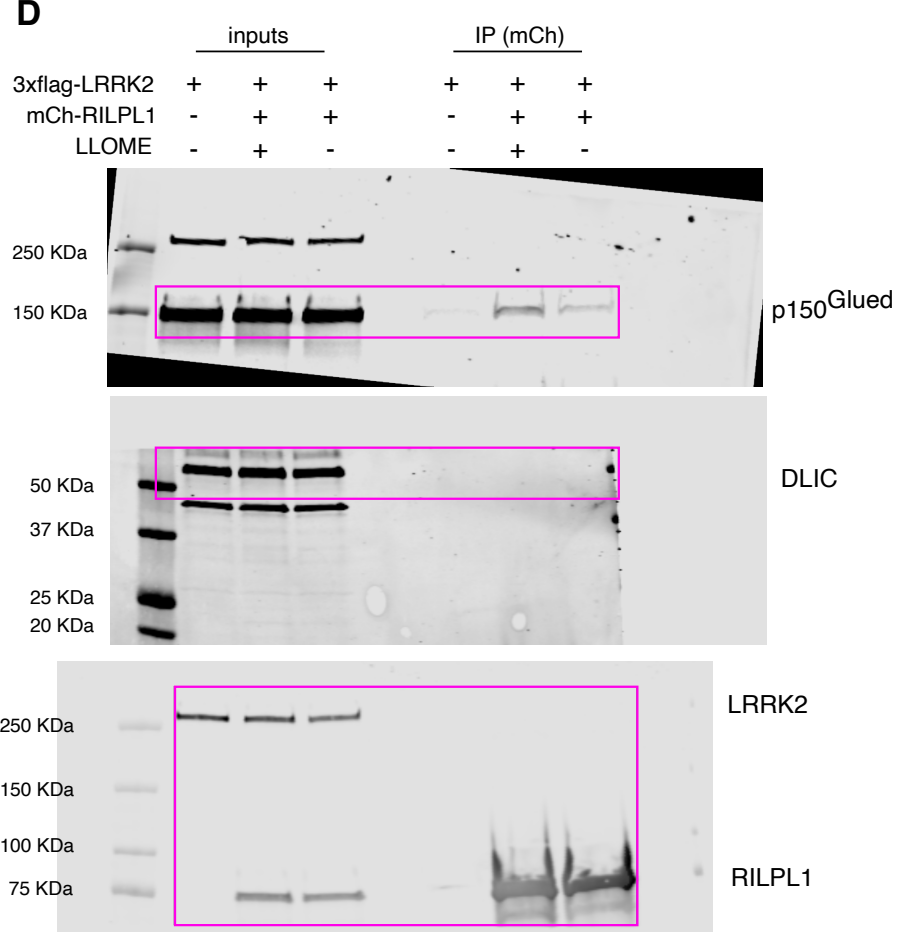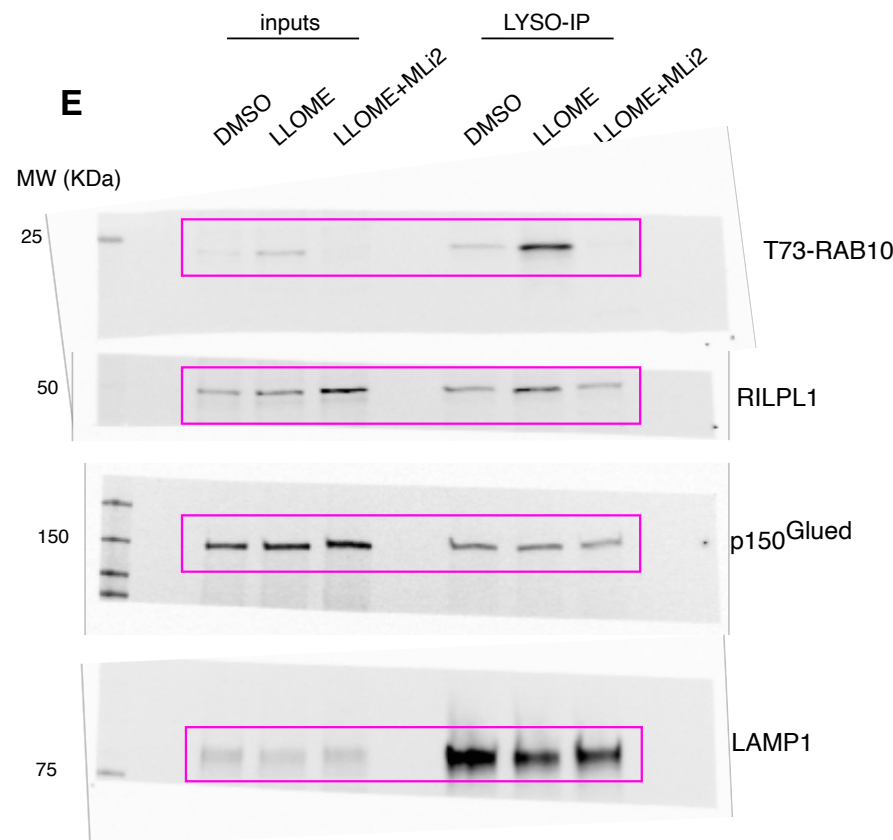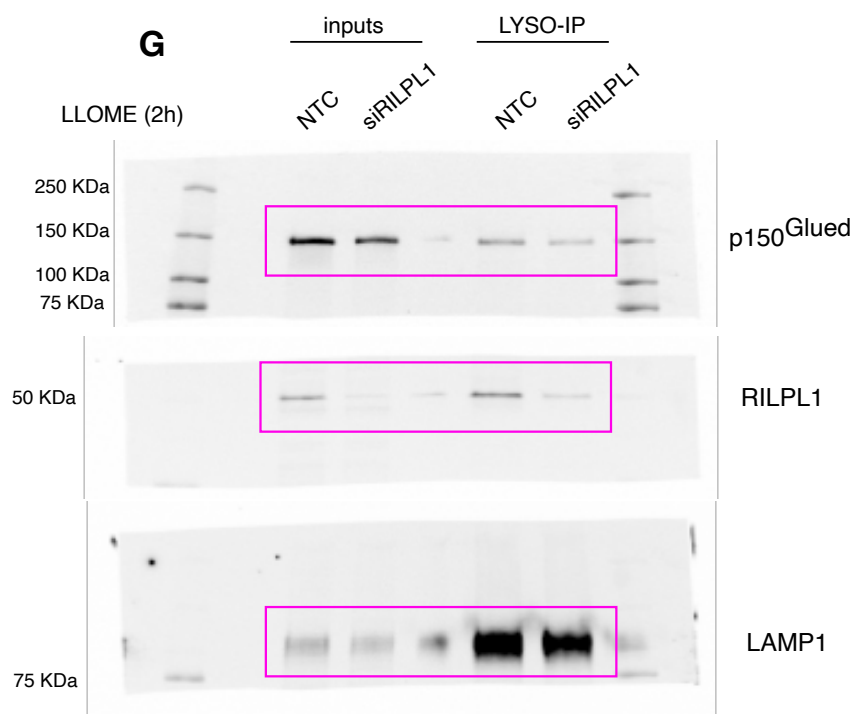

Supplement: SourceData F7 — is the source file for Fig. 7. [file jcb_202404018_sourcedataf7.pdf]

NTC

siJIP4

siRILPL1

I

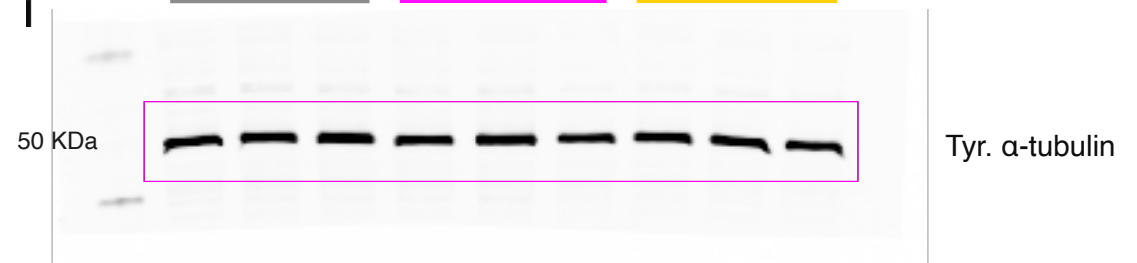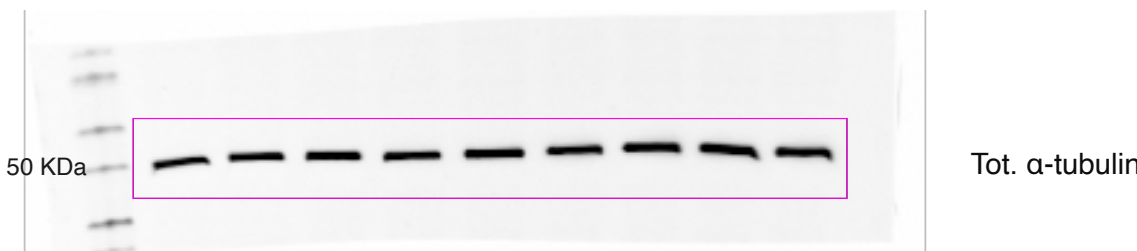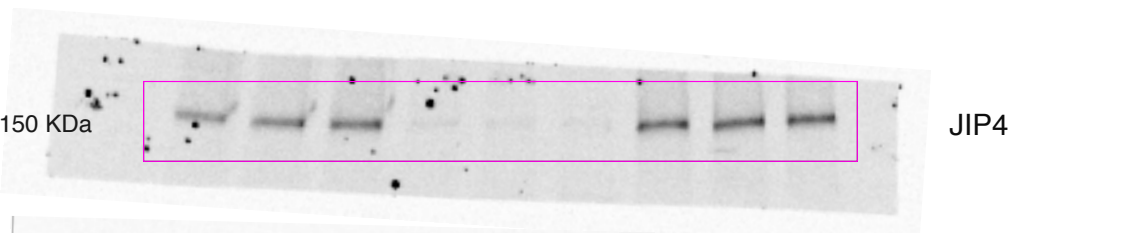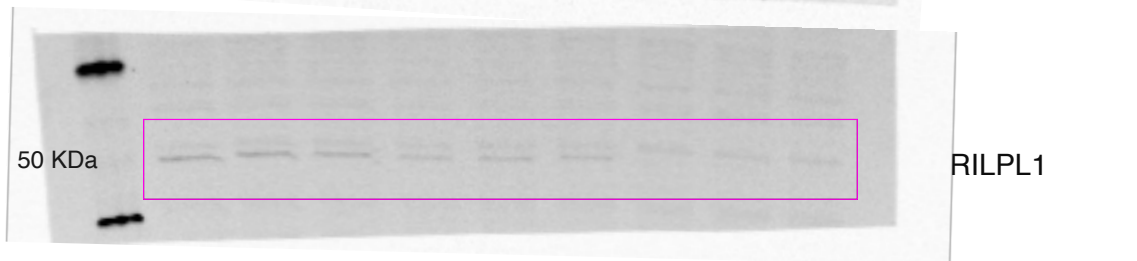

Supplement: SourceData F8 — is the source file for Fig. 8. [file jcb_202404018_sourcedataf8.pdf]

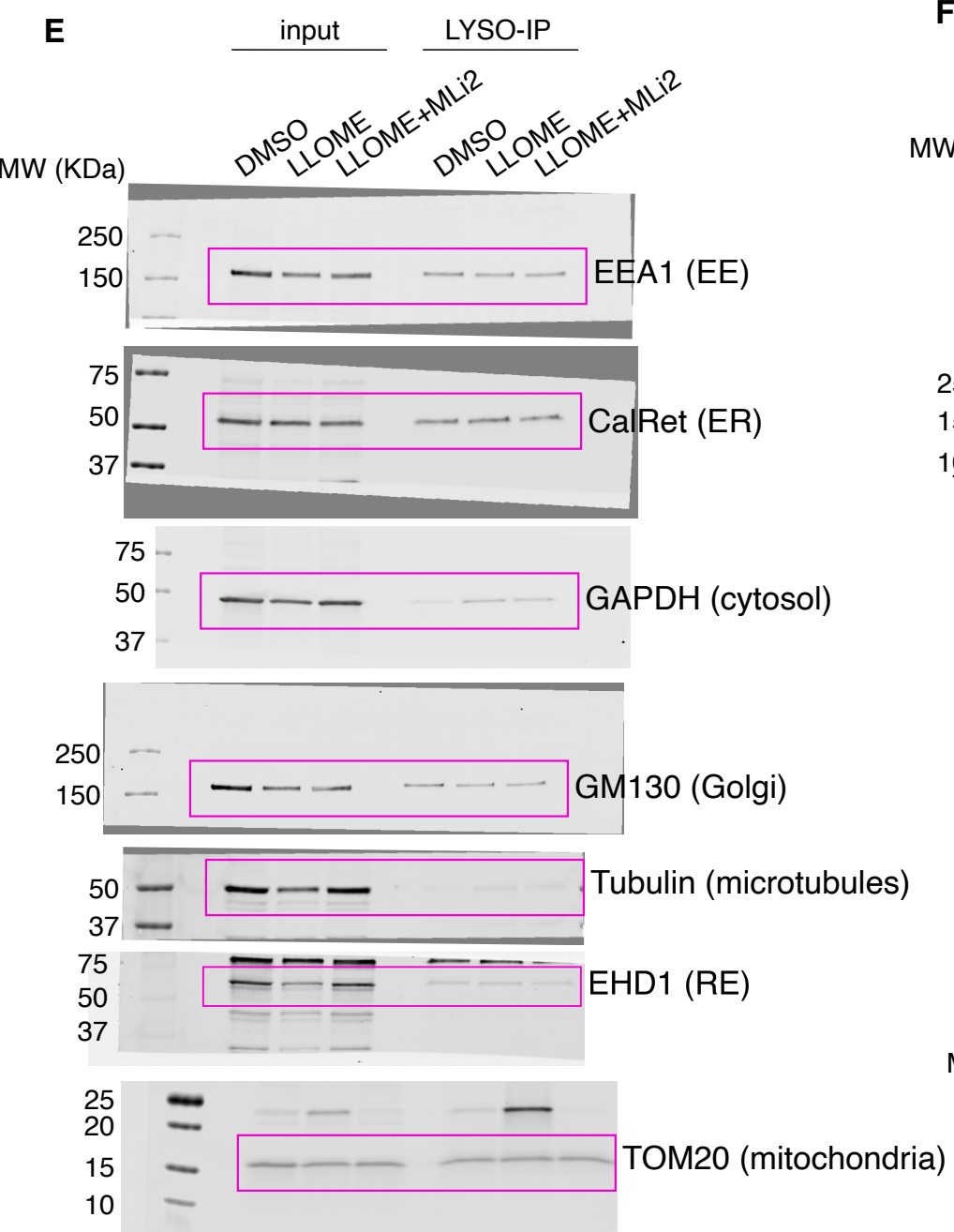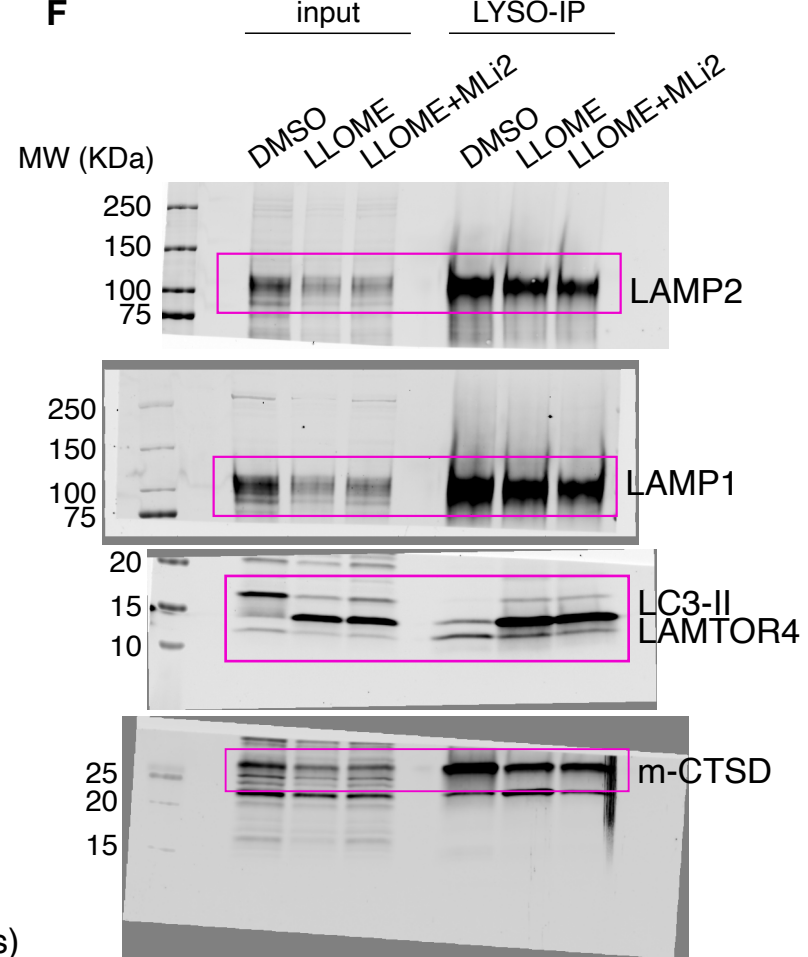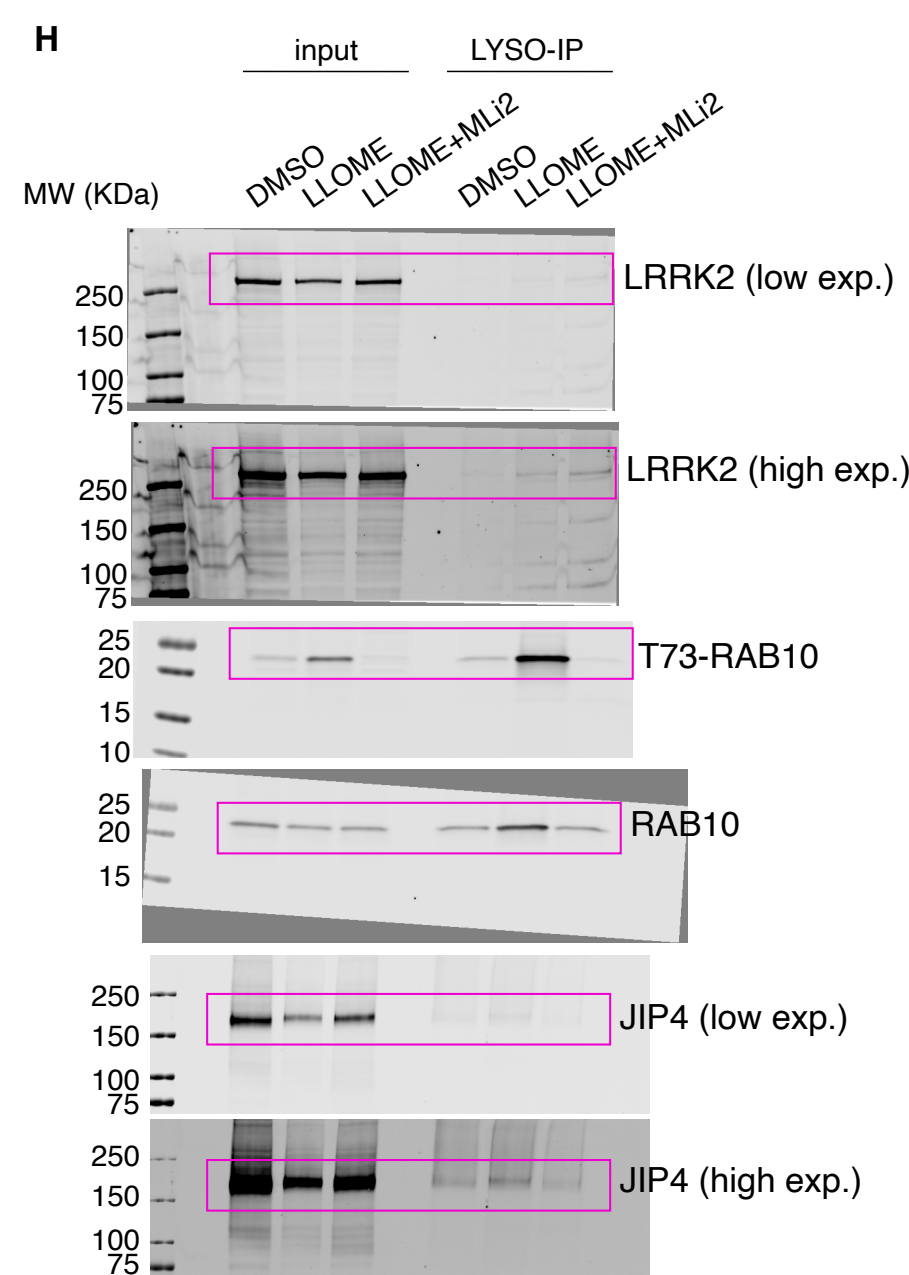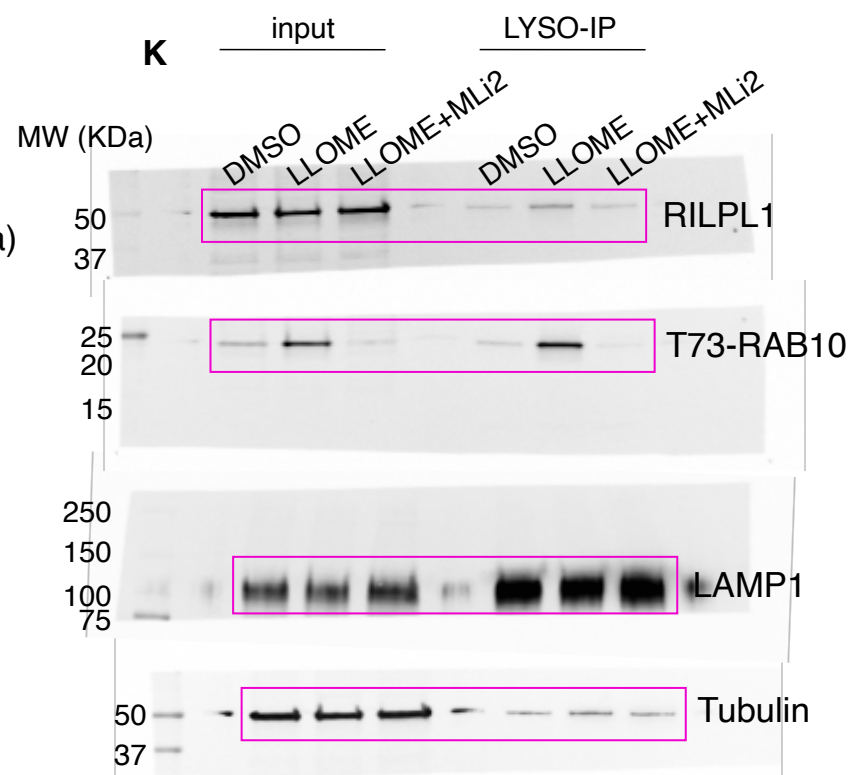

Supplement: SourceData FS1 — is the source file for Fig. S1. [file jcb_202404018_sourcedatafs1.pdf]

**A**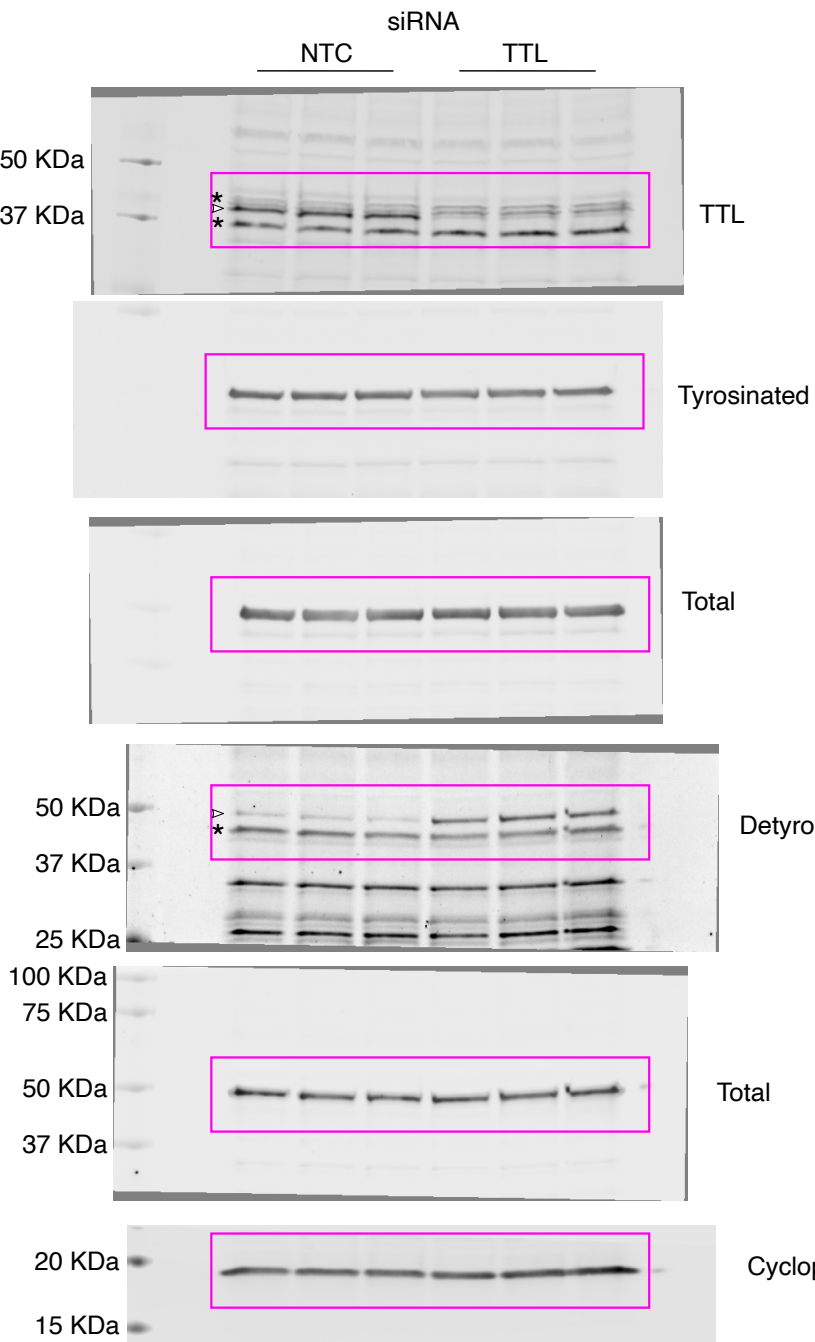**G**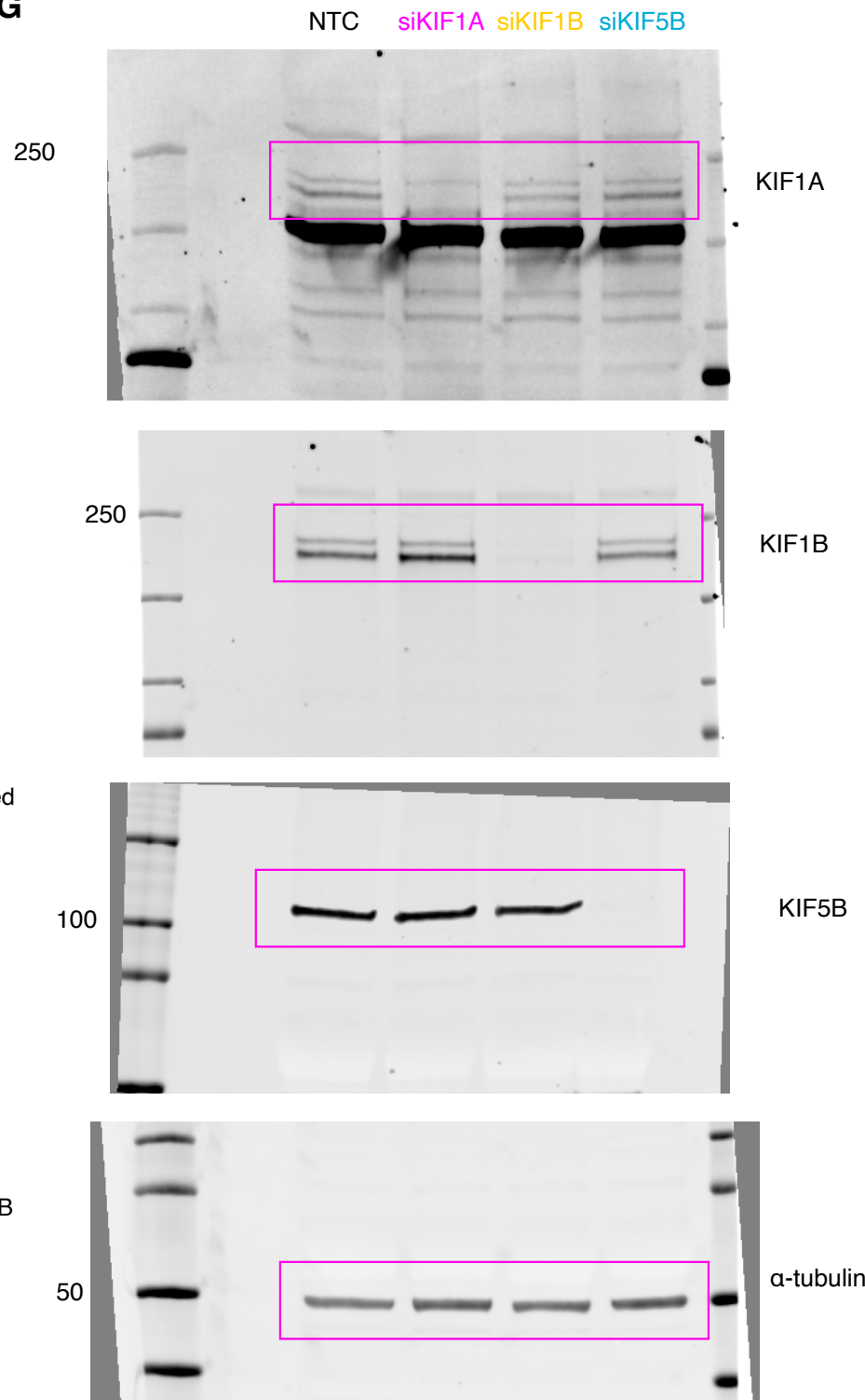

Supplement: SourceData FS5 — is the source file for Fig. S5. [file jcb_202404018_sourcedatafs5.pdf]
