# Supplementary material for: JIP4 and RILPL1 utilize opposing motor force to dynamically regulate lysosomal tubulation
Source: J Cell Biol. 2025 Sep 24;224(11):e202404018. doi: 10.1083/jcb.202404018 (PMC12459091; doi:10.1083/jcb.202404018)
Supplement: SourceData FS3 — is the source file for Fig. S3. [file jcb_202404018_sourcedatafs3.pdf]

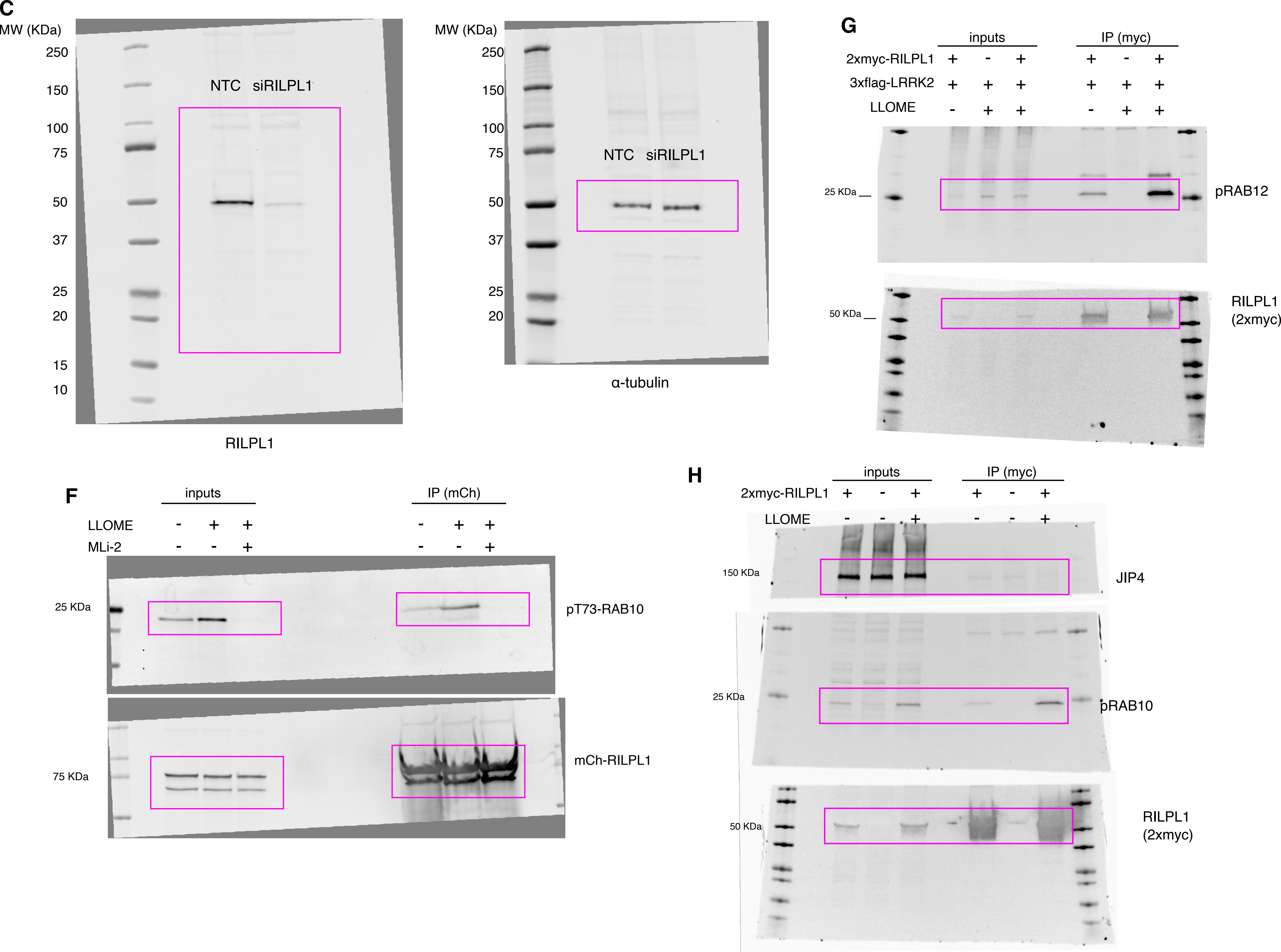
C
MW (KDa)
250
150
100
75
50
37
25
20
15
10
NTC siRILPL1
RILPL1
MW (KDa)
NTC siRILPL1
α-tubulin
G
inputs
IP (myc)
2xmyc-RILPL1 + - + + - +
3xflag-LRRK2 + + + + + +
LLOME - + + - + +
25 KDa
pRAB12
50 KDa
RILPL1 (2xmyc)
F
inputs
IP (mCh)
LLOME - + + - + +
MLi-2 - - + - - +
25 KDa
pT73-RAB10
75 KDa
mCh-RILPL1
H
inputs
IP (myc)
2xmyc-RILPL1 + - + + - +
LLOME - - + - - +
150 KDa
JIP4
25 KDa
pRAB10
50 KDa
RILPL1 (2xmyc)
